# Student perspectives on the value of rural electives

**DOI:** 10.4102/phcfm.v7i1.752

**Published:** 2015-06-26

**Authors:** Ian Couper

**Affiliations:** 1 Centre for Rural Health, Faculty of Health Sciences, University of the Witwatersrand, South Africa

## Abstract

**Background:**

Medical students in the Faculty of Health Sciences at the University of the Witwatersrand (Wits) in Johannesburg have the opportunity to do electives at the end of the first and third years of a four-year graduate-entry medical programme. Upon their return they are required to write a short portfolio report. Over the period 2005 to 2011, 402 students chose to do rural electives.

**Aim and setting:**

To understand the value of rural electives from the perspective of medical students in the Faculty of Health Sciences at Wits, as derived from their assessment reports.

**Methods:**

A review was conducted of 402 elective reports. Common themes were identified through repeated reading of the reports, and then content analysis was undertaken using these themes.

**Results:**

Major themes identified were the reasons for choosing a rural facility for the elective, including going to a home community; benefits of the elective, especially in terms of clinical skills and personal growth; relationship issues; the multiple roles of the rural doctor, who is often a role model working in difficult conditions; and the challenges of rural electives.

**Conclusion:**

The electives were overwhelmingly positive and affirming experiences for students, who developed clinical skills and also learnt about both themselves and their chosen career.

## Abstrait

**Les points de vue des étudiants sur la valeur des stages ruraux.**

**Contexte:**

Les étudiants en médecine de la Faculté des Sciences de la Santé de l’Université du Witwatersrand (Wits) à Johannesburg peuvent choisir de faire un stage électif à la fin de la première et troisième année du programme de quatre ans d’accès aux études médicales. A leur retour ils doivent rédiger un court rapport de portefeuille. De 2005 à 2011, 402 étudiants ont choisi de faire un stage en zone rurale.

**Objectif et cadre:**

Comprendre la valeur des stages en zone rurale du point de vue des étudiants en médecine de la faculté des Sciences de la Santé de Wits, à partir de leurs rapports d’évaluation.

**Méthodes:**

On a étudié les 402 rapports électifs. On a identifié des thèmes communs en relisant les rapports, suivis d’une analyse du contenu conformément à ces thèmes.

**Résultats:**

Les thèmes clés identifiés étaient les raisons du choix d’un établissement en milieu rural pour le stage électif, y compris la communauté d’origine; les avantages du stage électif, surtout du point de vue des compétences cliniques et du développement personnel; les problèmes relationnels; les rôles multiples du médecin de campagne qui est souvent un modèle travaillant dans des conditions difficiles; et le défi des stages électifs en zone rurale.

**Conclusion:**

Les stages électifs ont été des expériences très positives et mémorables pour les étudiants qui ont développé leurs compétences cliniques, et ces stages leur ont aussi enseigné des choses sur eux-mêmes et leur future carrière

## Introduction

Exposure of undergraduate medical students to rural health care during their training is recognised to be an essential strategy in the eventual recruitment of doctors to rural areas.^1-3^ Various models exist for this exposure, which include both compulsory and voluntary placements in rural health facilities and practices. The role that rural electives can play in encouraging students to consider rural practice, as well as in their general educational development, has not been explored in the literature.

The University of the Witwatersrand (Wits) in Johannesburg introduced a new medical curriculum in 2003, in the form of a four-year graduate-entry medical programme (GEMP). GEMP years 1 and 2 focus on problem-based learning in systems blocks, with weekly clinical skills and hospital exposure. GEMP years 3 and 4 consist of a series of clinical rotations in all major disciplines. Two elective periods are included in the programme: the first is a two-week period at the end of the first GEMP year, which may be undertaken anywhere in Southern Africa, whilst the second is a four-week exposure (that may be split into two two-week periods) at the end of GEMP third year, which may be undertaken anywhere in the world. Students are required to submit a two-page portfolio report reflecting on their elective experience, based on objectives set prior to the elective in consultation with a faculty supervisor, in order to sit the final examinations in the second and fourth GEMP years.

As part of its strategic goals, the Centre for Rural Health in the Wits Faculty of Health Sciences aims to recruit, educate and support human resources for rural health care. Through the academic division of Rural Health it offers the opportunity for students doing electives to gain rural experience, mainly in rural district hospitals, all over South Africa and even in other countries.

A steadily increasing number of students signed on for rural electives between 2003 (when GEMP 1 rolled out) and 2008, increasing from 5 (2.3% of the GEMP 1 class) to 85 (19.8% of the combined GEMP 1 and 3 classes), with a current steady state of 60–80 students (about 12% – 18%) signing up for rural electives. The initial increase in numbers, which occurred largely due to interpersonal communication, and the ongoing level of interest suggests that students were gaining something important through the rural elective experience.

### Aim and objectives

It was decided to ascertain what the value of these rural electives is in the eyes of the students themselves. A review was conducted of the student elective reports received over seven years from 2005 to 2011.

## Methods

A qualitative review was conducted of the portfolio reports submitted by students completing rural electives over the period 2005–2011. A total of 402 electives were undertaken. Of these, 371 were undertaken in rural public health facilities in South Africa, in all of the nine provinces, and 3 in urban district hospitals (due to last-minute difficulties encountered by students, such as lack of finances to travel); 13 were completed with rural private general practitioners in South Africa. Twelve students completed their electives in rural areas outside of South Africa, 11 of these in other African countries and one in the Northern Territory of Australia. Three students worked in non-governmental organisations, one of which was in Swaziland.

The majority of students (72%) completed these electives at the end of GEMP 1.

All 402 reports submitted over the seven years were included in the review. Common themes were identified through careful reading of the reports over the first five years. A content analysis of each report was then undertaken using these themes, to ensure that all major issues mentioned by students were covered, and copies of the reports were divided into the themes using cut and paste. The reports for the latter two years were reviewed in the light of the above themes; no additional themes were found, but relevant comments by students were added to the database.

Students are required in their portfolio reports to explain why they chose the particular site, what learning occurred and how this related to the objectives set with the internal (faculty) supervisor. These reports are assessed by this supervisor, and therefore reflections were likely to be more positive in nature, creating an inherent bias. However, this is mitigated by the fact that the mark has a negligible impact on overall grading – the main requirement is to submit a report. Furthermore, the reports are not seen by the external (host) supervisors, so honesty about the experience is also promoted.

### Ethical considerations

Prior ethical approval was obtained from the Human Research Ethics Committee of Wits. Reports were only reviewed after students had, at a minimum, completed their assessment process for the year subsequent to their elective, and therefore there was no possible influence on student outcomes.

## Results

The major themes identified in the reports are listed in Table 1. Quotations from the reports are used below to illustrate these themes.

**TABLE 1:** Themes that emerged from the reports of students who completed the rural elective.

Theme	Sub-themes
1.Reasons for choosing a rural hospital	a. Going home
	b. Experience of different culture
	c. Visiting beautiful places
	d. Gaining broad clinical experience
	e. Experience of less-resourced/poor area
2.Benefits of the elective	a. Clinical skills
	b. Personal growth
	c. Confirmation of chosen career
3.Relationship issues	a. Communication
	b. Teamwork
4.The rural doctor	a. Generalists
	b. Role models
	c. Negative examples
	d. Differences
	e. Working conditions
5.Challenges of the rural elective	a. Language barriers
	b. Cultural differences
6.Reflections	a. On rural medicine

### Reasons for choosing a rural hospital

Students offered a variety of reasons for choosing rural sites for their electives. For a number of these who came from rural areas, they were returning home, to the health facility closest to where they lived:

‘I did my elective at [*A*] Hospital. … Since it is my home hospital, I did not realise how “rural” it is because I am used to such an environment.’

‘I chose it because it is very close to my home [*about 20 min drive*] and from previous experience I knew that I would be able to do a lot of practical work.’

Even within the motivation of going home there were nuances illustrating different student needs and interests:

‘I thought it would be a good opportunity to get away from it all. I am from a small town in Mpumalanga. The city lights and all it has to offer holds no attraction for me. I miss home a lot, not only the veld or the simplicity of it all, but especially the people. The further you move out of the city the warmer the people become, when they ask you how you are, they really care.’

‘I actually wanted to have an exposure to the kind of service my family and my community gets.’

‘I spent the entire four weeks of my elective at the [*B*] Hospital, being my hometown, and also the place where I intend spending my future career. Just being able to treat patients who remembered me as a little boy growing up in front of them brought about a feeling not matched in my five years of training so far.’

Some students specifically saw the elective as part of the process of giving back to the community that supported them:

‘I wanted to know the health problems that my community was facing. It is a hospital that I have grown to love and I hope to find myself serving the community in that hospital one day. I was born in that community hospital and I plan to give the people in my community the best health care they need.’

‘I felt it necessary to give back to the rural community who had supported me during my high school years.’

For a few students there was an opposite reason – to get away from home:

‘I wish I could say that the reasoning behind choosing to do a rural elective was altruistic … The truth behind it is that choosing to do to a rural hospital meant a two-week holiday, away from home, all under the guise of “community service at a rural hospital”.’

Some students wanted to experience a different cultural milieu.

‘An elective in rural medicine in South Africa was a way for me to understand rural poverty and the pertinent issues at play in public health in developing areas.’

‘This was a chance for me to enhance my personal and professional development by understanding my culture better, then I could understand my patient's culture …’

Whatever other reasons there were, the opportunity to gain hands-on clinical experience was very important:

‘My main objective … was to do as many practical things as possible in order to prepare for my clinical years.’

‘I felt that I needed exposure to working in communities and dealing with common conditions.’

‘The reason for my choice was because I wanted to experience total medicine as well as go to a hospital where I would learn a lot. I think I made the correct choice because at the end of my two-week elective I felt as though I had learnt so much more than I expected to and was more passionate about wanting to be a doctor and work in South Africa than I have ever been before.’

Students recognised that this practical learning was different from what they were experiencing in the tertiary academic hospitals:

‘I also thought that it would be a great opportunity for me to learn new things as well as practice the skills and theory that I have been taught, since there wouldn’t be situations where a consultant, three registrars, two interns and four medical students are all crowding around one patient, as is sometimes the case at the hospitals we attend in Johannesburg.’

‘I chose [*C hospital*] because of the opportunity of non-competitive learning. Students are the most curious creatures and get tremendously fascinated by the new environment of medicine they find themselves in – filled with the abundant amenity of learning about the human being. Deliveries, lumbar puncture, pleural taps, anything, we jump at the opportunity to be the ones performing the procedures. But how often do you get to do these on a daily basis in a teaching hospital like [*Z*] Hospital, hardly.’

Part of the broader experience was also seen to be gaining an understanding of less-resourced or poorer areas

‘I had heard many stories about the facilities at rural hospital being inadequate, and living conditions being nearly as bad, thus it was with trepidation that I decided to step away from the comforts of urban life, and actually understand what the majority of South Africans deal with on a daily basis.’

‘I truly wanted to experience and understand the difficulties rural health practitioners face on a day-to-day basis, away from the busy and bustling resource-rich urban areas of South Africa.’

This included a desire to compare rural and urban hospitals:

‘to see the difference existing between rural and urban settings.’

‘I needed to understand the community better and also be exposed to the rural health setting that is so prevalent in this country. It would be naïve of me to assume that work environment in the big city hospitals is a reflection of the many smaller hospitals serving the bulk of the nation.’

### Benefits of the elective

#### Improvement in clinical skills

Students identified a range of important benefits from doing a rural elective. Most significantly, all students reported improvements in their clinical skills. A number of reasons were given for this. Firstly, there was greater independence and responsibility:

‘I was following up patients, not just any random patients but “my own patients”.’

‘Due to the massive doctor shortage, I was able to get heavily involved in a wide range of activities at the hospital that most third-year students of medicine would never be able to participate in.’

There was the opportunity to put theory into practice, which was valued especially because it allowed students to realise how much they had learnt:

‘Though initially nervous, I surprised myself with my own ability as I became more confident in applying the theoretical knowledge and clinical skills I had acquired at Wits through the year.’

‘It was so encouraging to realise that we have learnt a great amount this year, as we could start to ignite our own reasoning processes using the facts that we have accumulated through GEMP 1. Being able to pull the basic sciences, clinical skills and patient doctor and community doctor themes all together into one reality of practicing medicine was very stimulating.’

Students developed a wide range of clinical skills as a result of ‘learning by doing’:

‘This elective gave us the unique opportunity to *do* rather than observe.’ [*Student's emphasis*]

‘It was a wonderful opportunity to hear different heart and chest sounds and practice the clinical skill we have learnt through the year. … Now I finally know what crackles are meant to sound like!’

‘We got hands-on experience that every medical student craves from the first day of registration.’

This was enhanced by local supervising doctors who ‘were also very helpful when it came to my learning’:

‘I appreciated the care and time the doctors took in explaining and teaching different theory and skills to me.’

‘We could experience all the challenges of dealing with a wide spectrum of patients, from first encounter to discharge, but all the time we had a safety net – we could always ask for help or advice if we were uncertain what to do.’

For some the process gave them a new appreciation of their medical school training:

‘Another important thing I realised is that the GEMP programme is not as bad as I thought. It is actually a good programme compared to the programmes offered in other universities. I realised this when I met medical students from other medical schools who seemed not to know a lot and had no idea of some of the models.’

However, they also discovered gaps in what they are learning:

‘You learn a lot about yourself and you learn an approach to disease unlike what is taught in the syllabus …’

#### Personal growth

Personal growth was identified as another major benefit of the rural elective experience, and was described in a multitude of ways. Much of this was about learning aspects of medicine outside of the curriculum:

‘I came to realise that getting 90% in the exams won’t make me a good doctor but will make me pass and get promotion. But the real world out there in the hospitals is all about helping patients, saving lives, handling sensitive issues and understanding all your patients and their needs. This elective made me to consider my own fears, likes, dislikes, this will help me in choosing my area of specialisation in future as a doctor.’

‘One of the most profound realisations that I had whilst on my rural elective, was the need for inward reflection. Throughout the academic GEMP 1 year, it becomes easy to place all one's focus on the theoretical studies, and forget about analysing how certain experiences affects one as a doctor and an individual. I never thought that my feelings and beliefs could be influenced by interacting in the consultation, believing that I could focus only on my skills and knowledge.’

‘I did not expect to come out of the elective with a better understanding of who I am, and a large amount of personal growth.’

Aspects of professional life and coping with being a doctor were important:

‘I learnt how to handle pressure at work and also to discuss sensitive issues. I then realised that you don’t need books to study how to handle pressure at work. This comes from you as an individual.’

‘I came to realise, and I hope that I never forget this, that as a doctor it will never be my place to judge people for anything they do, but rather act compassionately and professionally no matter the circumstance.’

‘My elective revived my faith in medicine. It made me think. It made me re-evaluate my reasons for studying medicine. It made me realise that in a cold world and an icy profession there is still love and the warmth in caring.’

Some students described the experience as life-changing:

‘It started out as a challenge of surviving two weeks in a rural setting and ended up a life-changing experience.’

‘I was not the same I was before I went and I will be forever changed by it.’

‘I believe I learnt the most important lessons in life, of which one of them is the fact that we need to see “people” before we see “patients”.’

‘Somewhere in the middle of rural KZN [*KwaZulu-Natal province*], when I thought I had lost everything, I found myself. I found who it is I’m meant to be. I found who I truly am. For that, I will be forever grateful.’

Being alone and outside of their comfort zone was certainly a factor in this growth:

‘Sure everyone says you “grow” on electives, but I had first-hand experience of growth. I firmly believe that being alone strengthened me tremendously. I found I could work alone, ask as many questions as I wanted to, go anywhere.’

‘Having entered medical school in my sixth year of tertiary study, I quickly realised that professional and indeed personal development only occur by continuously seeking alien situations, which provide opportunities to challenge oneself.’

For some students personal growth occurred in facing the reality of medical practice in South Africa for the first time:

‘I have been well aware of how big a problem TB [*tuberculosis*] and HIV [*infection*] present but being confronted with the human face of the disease every day in the volumes we were, really brought home the magnitude of the problem. The feeling of helplessness weighs heavy, but on this background the moments of hope, painted in such stark relief, stand out most boldly.’

‘I realised that as a doctor I will have to face crises and deal with losing patients and I needed to have adequate coping mechanisms to get me through these dark times and prevent burnout.’

‘… by the end of two weeks, I was accepting limitations (power cuts, limited time with patients, etc.) such as these far more easily and focusing on the solutions to the problems, rather than on the problems themselves.’

‘This was the first time, I feel, that I was truly exposed to the personal lives of people who visit the hospitals. I have never felt such anger as I felt when I saw a nine year old girl who had been abused and actually prostituted by her family.’

#### Confirmation of their chosen career

Another benefit expressed by many students was a sense of confirmation of their chosen career:

‘Not only has this elective experience confirmed my aspiration to become a doctor but it also has opened my eyes to other hospitals in this country and how they work.’

‘My enthusiasm [*was*] rekindled in the exciting medical field, not only by all the practical experiences I have had but also by the spirit of the wonderful people and patients I have met.’

‘I learnt a lot in so many ways, clinically, culturally, personally and spiritually. It definitely also encouraged me as a medical student, [*to*] study hard, not necessarily only for myself, but also for the patients that I will one day have as a qualified doctor.’

### Relationship issues

Students learnt a lot through working together with doctors and other health professionals, particularly in terms of communication and team work.

#### Need for good communication

Good communication and positive working relationships were frequently noted as important:

‘It made me realise that having good relationships with other people is very important.’

‘My elective was a positive experience that taught me the value of working with the people in the community including the staff of the hospital who were part of that community.’

This included good communication with patients:

‘For the first time I felt as though I had the opportunity to truly understand the doctor-patient relationship and its significance in assuring quality health care.’

‘Even though I tend to be shy and battle to talk to people I do not know, I started really enjoying listening and talking to patients and even tried to speak some Zulu, which made them feel much more comfortable and willing to share their experiences.’

Sometimes the lessons were learnt from poor examples:

‘In the consultation the doctor could make the person feel like a nuisance or better for just being there, simply by virtue of their attitudes. I saw patients being dismissed and ignored and not listened to.’

‘It is evidently clear that poor relationship between health care workers and patient has a detrimental effect on the general services delivered by the hospital.’

#### Importance of teamwork

Many lessons on the importance of teamwork were presented:

‘One of the most valuable lessons I learned is how important teamwork is in the hospital setting. As one nurse told me: “We work hard in this hospital because we all care for each other”.’

‘It was here that I learned the value of teamwork as doctors of various departments, physios, nurses and counsellors worked hand in hand in the management of patients.’

‘Team work is vital … I came to realise that as a doctor I would be incapable of fulfilling all the patient's health care requirements and depend on other disciplines for their expertise.’

Students came to realise that it is people that make the difference to health care:

‘… my biggest lesson: Infrastructure and equipment is important, but they don’t define a health facility, nor do they predict the success of that facility. Success is determined by the *people* who work in that facility.’ [*Student's emphasis*]

### The multiple roles of the rural doctor

The opportunity to work very closely alongside rural doctors provided insight into their roles. Rural doctors were seen to be capable generalists, who need to deal with a wide range of problems:

‘To put it bluntly, rural doctors are just generally more capable and in control than urban doctors.’

‘It amazed me that a rural doctor is able to treat all the signs and symptoms so effectively and treat every system of the body, even though he is just a general practitioner.’

‘They walk in each morning and their job description is not “the surgeon” or “the physician”. They are doctors who just have to deal with whatever gets thrown in their face.’

‘It's not just about working in the hospital, but it is also about being an active member of the community.’

Rural doctors were also seen to be role models and mentors for the students:

‘One of the doctors practises medicine in a way that I would like to be able to do one day soon – with knowledge, skill, compassion, and love while at all times respecting the patient and being realistic about my own limitations.’

‘One ward round stood out particularly in my mind, with Dr [*M*], who was a caring, gentle, humble man. The mutual respect and the dynamic working relationship between the Dr and the nurses were awe-inspiring. Dr [*M*] greeted each patient by name, asked her how she was and inquired if their families had been to visit them the previous day. At the end of his instructions to the nurse, he added “Also, give a liberal dose of TLC”. The genuine interest and care of Dr [*M*] for his patients set an example for me as to what kind of doctor I would like to be one day.’

Even with their imperfections, doctors could still inspire students:

‘This was the most inspiring of the elective, that the doctors, although overworked and underpaid and sometimes lost it with the non-complaint patient, they put patients as top priority.’

However, a number of students also encountered negative examples:

‘I do not ever want to be a Dr like the Dr that was on call that night. Imagine – he caused a baby to die because he did not show up when he was on call. If I ever become a Dr like that, I would want to be shot … how can a person call him or herself a Dr and then be so negligent?’

‘One of the two doctors assigned to work once a week at the clinic … has a mega attitude, does not exam patients, does not talk to the nurses and chooses who he wants to work with.’

Students learnt that all doctors are different, and that they need to choose what kind of doctor to be:

‘I found [*E*] Hospital to be a place of great compassion, dedication and excellence, but also a place of apathy and desolation … There are those individuals who approach situations seeking excellence, those individuals who are always challenging themselves to do better and those who choose a path of apathy and discontent that ultimately results in poor service. I … will use my experience as the basis to continually evaluate the effort I put into and quality of my service to patients.’

#### The working conditions of rural doctors

The working conditions of rural doctors made a great impression on students. Multiple challenges faced in terms of resources were described:

‘The biggest barrier is the lack of a transport system for patients. We referred to hospitals that were hundreds of kilometres away and there were no arrangements made for patients to be picked up or dropped off.’

‘At this under-resourced, understaffed and overburdened hospital, patients have gotten used to waiting an average of 12 hours to be seen. Walking past the waiting room, I saw people with broken limbs and chronic illnesses almost cussing at the arrival of the paramedics because they know that an emergency meant that they will have to wait a few more hours.’

Facilities were, however, noted to be variable:

‘From a resource allocation point of view I was very very impressed: the hospital had supplies in abundance; the supply cupboards were always fully stocked with a variety of items, so treatment of a patient was never compromised because a needle was too big, or the plaster of Paris was too small.’

‘Patient care was seriously being affected by this poor infrastructure. Patients couldn’t be admitted in the correct wards because of a lack of space and beds. The doctors were under too much pressure and overworked also decreasing quality patient care.’

‘The most striking thing about the hospital's impression was how clean and organised it was. It almost seemed like a private hospital. It gave me hope that it really wasn’t impossible to keep a hospital clean. It takes dedication, good management and team work, but it sure is possible.’

### Challenges of the rural elective

Students highlighted particular challenges, in addition to those mentioned above. Language barriers were often raised as an issue:

‘Communication was an enormous predicament. It was very difficult to come across a person who could actually speak English, so most consultations were done with a nurse on hand.’

‘Out in the community I realised the absolute barrier that language could be. Many times I sat like an idiot nervously smiling at the patient when the doctor and translator had gone. From this I realise that I desperately need to make a plan to learn an African language if I ever hope to serve in a community like this.’

‘It was a challenge for me to work with an interpreter. I often felt that my questions were not asked sufficiently and the interpreters were having private conversations unrelated to the topic at hand.’

Students also noted cultural differences as potential barriers:

‘I learnt a lot about the history, cultures and way of life in Africa's Eden.’

‘The cultural barriers were as frustrating as ever.’

However, these were also great opportunities for learning:

‘As well as diagnostic and clinical skills learnt, there were many other lessons that I learnt … Some of these included valuable lessons on the Xhosa culture. The Transkei is very much dominated by the Xhosa people of South Africa which has its own culture very different to my Western ways. Some of these lessons included not [*to*] look/stare at patients directly in the eyes and that you should always greet people!’

‘This inspired me to learn some Zulu and I picked up practical phrases on my elective.’

### Reflections on rural health care

Students expressed their opinions about a number of issues, but in particular about rural medicine, and rural hospitals. Students argued for the value of rural exposure, for themselves and others:

‘I think that as a future doctor it is important to experience rural medicine as it forms an integral part of our country's medical need. We need to be able to interact with people of different cultures and different perceptions of health. This will allow us to see the challenges faced by rural doctors so that we may become adequate doctors who are better equipped at providing holistic and effective treatment to our patients.’

‘I did learn a lot but my question is that what do I (we) do to improve the health service of our rural hospitals? I think that I am the answer of this question. If we mobilised government to provide adequate resources and enough staff and create jobs, empowering people with education that might help.’

‘I am so glad that I decided to go rural for my elective and really think that every student should at least consider it for maximum return from their elective.’

Nearly all the students came away with a very positive feeling about rural health care, despite the problems encountered:

‘I really did find it amazing that despite the setbacks, the doctors were coping and the hospital was running smoothly. It is fantastic to see how much can be done with so little.’

‘If I were to sum up my time in Lesotho in one sentence it would be: “The happiest 10 days of my life”.’

‘After working [*in a rural hospital*], a doctor will be able to handle any other hospital setting.’

‘Rural health is amazing and my two weeks elective just made me realise the need of health practitioners in my hometown.’

## Discussion

Medical students at Wits have the opportunity to do a rural elective placement, an option chosen by a substantial minority of students. The reports of those students who chose to do the rural elective offer a fascinating perspective on the importance of this experience. Whilst these reports were submitted for assessment, which introduces bias, they do indicate the value of the rural elective experience.

The term ‘elective’ refers to a range of different curricular elements, with a common factor of student choice;^4^ in fact, in the United Kingdom the only essential requirement of a period of study defined as an elective is that students choose the clinical experience they have and set their own learning objectives,^5^ which is how electives are also viewed in the Wits programme. Usually electives involve short attachments outside of their immediate study environment, and most have a clinical focus.^4^

The range of reasons for choosing an elective site suggest that, whilst maintaining a clinical focus, diversity and complexity should be welcomed. It is very encouraging that many students go back home, as this portends well for future practice, according to international literature on retention,^1^ confirmed by local research.^6,7,8,9^ The fact that students are open to persuasion indicates an opportunity to use electives as a recruitment tool for rural practice. Whilst it is recognised that electives may be seen as tourist opportunities by some students, which will have negative influences on learning outcomes and on students’ attitudes,^4^ there was no evidence to suggest that this was the case amongst the reports reviewed, even in cases where students frankly described a beautiful environment as a key factor in their choice.

Rural training placements are frequently described as positive learning experiences by students and preceptors.^10^ Such rural experiences not only impact on student learning and the development of clinical skills, but there is also evidence of that rural training influences future practice site and career choice.^10^ Whilst this study cannot offer any evidence of this, the impact described by students and the personal growth reflected in their portfolio entries is indicative of a significant effect, and electives in international health – many of which are in rural and underserved areas – have been shown to influence career choices.^11,12^ Whether or not it influences future practice location, the electives motivate students with regard to medicine as a career in general.

Students’ willingness to explore and learn about issues such as rural health care, working in under-resourced areas, and the challenges of poverty is encouraging. This opportunity for students to explore the possibility of a future career in rural medicine in a relatively low-stress situation fairly early on in their clinical careers is important.

Developing clinical skills was noted as the most significant benefit of the elective reported by students. This is commonly described as a key positive feature of rural training^13^ and of international electives.^11,12^ What is clear is that this broad clinical experience and personal growth, which was obviously also critical for many students, occur side-by-side, making the learning more holistic than usually experienced in traditional rotations. The clinical skills development is a major pull factor in students seeking to do rural electives, but the enthusiasm of their colleagues in preceding years arising from the personal growth reported is no doubt also a motivator. Whilst there were students who encountered negative examples and were distressed by individual experiences, it seems these did not detract from their overall learning and development. These factors alone make a strong case for extending this experience.

Students spontaneously wrote about personal growth and reflected on their experiences in relation to their present and future situations; such reflection may be important in developing social accountability amongst students.^4^ The level of reflection also demonstrates the connection students were able to make between theory and practice, which is often not possible to achieve within a classroom environment or even a pressurised academic hospital rotation.

A literature review of international health electives, most of which are not dissimilar from the Wits rural electives, indicates that these provide opportunities for medical students to strengthen existing skills or learn new diagnostic skills, with a focus on history-taking and clinical reasoning, and to practice medicine with fewer resources,^11^ which is what our students also report. This review also found that participants in such electives were generally more likely than non-participants to report attitudinal changes, such as greater appreciation of the importance of cross-cultural communication, public health interventions, and providing care to the underserved,^11^ which also accords with this study. In addition to these features, another study in the United States of America (USA) found students (like ours) also reported heightened awareness of social determinants of health, increased self-awareness and greater insight into the human side of medicine.^14^

Students express an interest in becoming rural doctors and doing rural medicine. How much this is presented for the sake of the report and in the emotion of the moment is unclear. Long-term follow-up would be needed to determine this. No doubt further nurturing of this interest would be required to make a difference. Preceptors are often important role models, and have been shown to have a very strong influence on future career choices.^15^ However, simply having a strongly positive attitude towards rural health care, regardless of future practice location, is already an important movement. For a few perhaps the elective was the life-changing moment that some claimed it to be. If the elective did nothing other than affirm students in their choice of career and rekindle their determination to succeed in their studies, as expressed by many, then it surely still had an important role.

It is encouraging for Wits that students felt that the GEMP programme gave them a good preparation for the practical work in a rural hospital, and that they felt better prepared compared to students from other universities. However, many students suggested the need for an African language course to facilitate doctor-patient communication.

The limitation of this study is the use of reports that have been submitted for grading and assessment, which does bias the kind of input that students give, as noted earlier. However, the numbers involved and the consistency of the information obtained from students in many different sites, over two curricular years and seven calendar years, suggests that the themes do in fact closely represent the reality of the rural elective experience for our students.

Possible future research includes interviews and skills audits with students after rural electives (immediately and after a year or more), as well as interviews with host supervisors.

## Conclusion

Whilst it is important continually to review the value of electives, it is clear that students completing rural electives at Wits are benefitting greatly from the experience. A review of the fourth-year curriculum across medical schools in the USA indicated that elective experiences commonly duplicated other experiences and did not enhance student education;^16^ the evidence from these student reports is in stark contrast to that.

In conclusion, the rural elective was overwhelmingly a positive experience for the students. They learnt a great deal about themselves as well as developing their clinical skills. The elective was an affirming experience for most, which gave them confidence in their clinical skills and their chosen career.

Who can argue with a student's perspective that ‘the most important lesson I have learnt is to love what I am doing’? This would argue for consideration to be given to making a rural elective a required component of the curriculum, which also has the potential to address the ongoing medical workforce shortage in rural South Africa.
